# Tissue Engineering Strategies for Intervertebral Disc Treatment Using Functional Polymers

**DOI:** 10.3390/polym11050872

**Published:** 2019-05-13

**Authors:** Youngjoo Choi, Min Hee Park, Kangwon Lee

**Affiliations:** 1 Department of Transdisciplinary Studies, Graduate School of Convergence Science and Technology, Seoul National University, Seoul 08826, Korea; choi.yj@snu.ac.kr; 2 Center for Convergence Bioceramic Materials, Korea Institute of Ceramic Engineering and Technology, Cheongju 28160, Korea; 3 Advanced Institutes of Convergence Technology, Gyeonggi-do 16229, Korea

**Keywords:** tissue engineering, functional polymers, intervertebral disc, degenerative disc, regeneration

## Abstract

Intervertebral disc (IVD) is the fibrocartilage between the vertebrae, allowing the spine to move steadily by bearing multidirectional complex loads. Aging or injury usually causes degeneration of IVD, which is one of the main reasons for low back pain prevalent worldwide and reduced quality of life. While various treatment strategies for degenerative IVD have been studied using in vitro studies, animal experiments, and clinical trials, there are unsolved limitations for endogenous regeneration of degenerative IVD. In this respect, several tissue engineering strategies that are based on the cell and scaffolds have been extensively researched with positive outcomes for regeneration of IVD tissues. Scaffolds made of functional polymers and their diverse forms mimicking the macro- and micro-structure of native IVD enhance the biological and mechanical properties of the scaffolds for IVD regeneration. In this review, we discuss diverse morphological and functional polymers and tissue engineering strategies for endogenous regeneration of degenerative IVD. Tissue engineering strategies using functional polymers are promising therapeutics for fundamental and endogenous regeneration of degenerative IVD.

## 1. Introduction

Low back pain (LBP) is highly prevalent today and is one of the major causes of disability; most people with LBP have to live with the pain throughout their lives. In most cases, LBP is related to intervertebral disc (IVD) degeneration that leads to neurological deficit, and disabilities of various other kinds. The number of people with back or neck problems increased from 20.7% to 24.7% of the US population from 1997 to 2005, and LBP was ranked number one in 2010 [1,2,3]. The total medical expenditure related with spine problems increased by 65% (adjusted for inflation) of the overall health expenditures [1,2,3]. The burden of pain, disability and societal costs caused by LBP is still growing especially in other developed countries.

**Figure 1:**
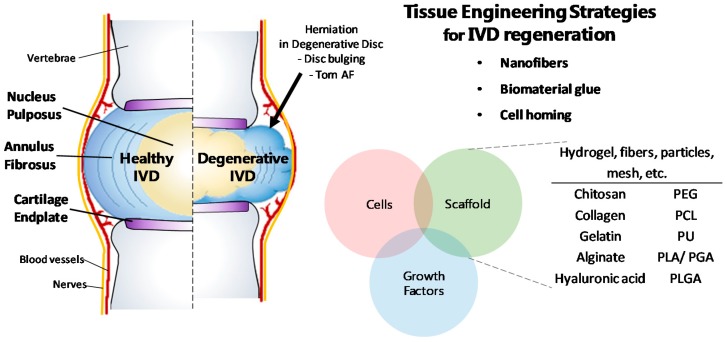
Features of human intervertebral disc (IVD) structure, and regeneration strategies for degenerative IVD using functional polymers in tissue engineering. Polymers are used as scaffolds in various forms such as hydrogel, fibers, particles, and mesh. Abbreviations: AF, annulus fibrosus; IVD, intervertebral disc; PCL, poly-ε-caprolactone; PEG, polyethylene glycol; PGA, polyglycolic acid; PLA, polylactic acid; PLGA, poly (lactic-*co*-glycolic acid); PU, polyurethane.

The IVD (Figure 1) is a fibrocartilaginous tissue placed between the vertebrae of the spine. Due to its avascular feature, IVD has a low concentration of nutrients and oxygen as well as low pH condition [4,5,6,7,8]. The concentrations of oxygen and nutrient decrease as the area is closer to the center of the IVD where fewer cells present compared to that in other tissues [5,6,7]. In the center of the IVD, nucleus pulposus (NP) that contains high water and proteoglycan contents is located and surrounded by the annulus fibrosus (AF) which is a multi-lamellar structure comprised of water and collagen [9,10,11,12,13]. The NP and AF are attached to the vertebrae by a cartilaginous layer named as cartilage endplate (CEP) [14,15]. The NP, AF, and CEP work together enabling the spine to move steadily and flexibly by bearing multidirectional complex loads of high compression, bending, and torsion [16,17,18].

The IVD diseases result from various causes such as aging, wrong posture, physical trauma, or genetic factors. Furthermore, the structural change and degeneration of IVD leads to a wide range of pain and other symptoms [8,19,20,21,22]. IVD degeneration affects spinal alignment, flexibility, or neural anatomy, and these effects cause degeneration of the IVD and spine leading to diseases such as herniation, CEP calcification, osteophytosis, stenosis, spondylolisthesis and spondylosis [23]. Many therapeutic methods to restore degenerative IVD were developed and invasive surgery was usually done to repair degenerated IVD in the past. General surgical procedures include fusion, disc replacement, and discectomy that subsequently evolved into microdiscectomy surgery which is less invasive than other surgeries for degenerated IVD [24,25]. Despite the efforts to repair degenerated IVD, degenerative disc disease has not been fully understood and limitations of the invasive therapeutic methods remain [3]. In order to overcome these limitations, therapeutic strategies for tissue and biological aspects have been emphasized. The eventual goal of IVD therapy should be endogenous regeneration within the degeneration site rather than surgical operation for temporary relief. Therefore, many studies recently tried to mimic this natural healing system through tissue engineering to regenerate defect sites using biomaterials, genetics, cell biology, and tissue morphogenesis [26]. Here, we review recent advances in tissue engineering strategies for IVD repair and regeneration using a variety of functional materials and methods using hydrogel, particle, and fiber. These strategies employing functional polymers have promising potential to treat degenerative IVD through endogenous regeneration and natural healing.

## 2. Background: IVD Structure, Function and Degeneration

In tissue engineering, an understanding of IVD structure and its biological properties is important to develop therapeutic strategies against degenerative disc disease. This is because tissue engineering for regeneration of the IVD is achieved by mimicking native IVD biologically and mechanically. The IVD is comprised of NP, AF, and CEP, and the three substructures work together to help the IVD to function [4].

### 2.1. Nucleus Pulposus

The NP is a gelatinous and hydrated tissue in the center of the IVD surrounded by multi-lamellar ring shape of AF [11,12,27,28]. Water that comprises 70–90% of NP is bound by negative charge of sulfate in proteoglycans, which makes NP have a high affinity for water and high osmotic pressure [9,10,29,30,31]. As the age increases, the weight of proteoglycan inside NP decreases to 30% and the water content decreases to about 70% [9,32]. Collagen type II fibrils and some collagen type III are the main collagen that accounts for 15% of the dry NP weight [9,29,33]. Collagen type II enhanced resistance to swelling, and collagen type III surrounds the cells in the NP and AF [33]. Multi types of collagen with three-dimensional fiber network constructs the NP matrix [34]. NP absorbs compressive forces and disperses loads from physical activities to other body parts through osmotic pressure [27,35]. In addition, hydrostatic pressure in an outward radial direction accommodates compression loads and generates tension in AF without collapsing [35,36]. In a healthy IVD, pressure of NP is 0.3–5 kPa in unconfined and 0.12–1 MPa in confined condition [37,38].

In NP tissue, cells are derived from the notochord, and these notochordal cells which are large and vacuolar throughout life [39]. However, as the NP matures, a population of round and chondrocyte-like cells increases and expressed marker changes [40]. The NP cells are present at a low density of 2–5 × 10^6^ cells/mL when the IVD is matured, and the NP cell population decreases with aging [39,41]. Adult NP cells have phenotype and morphology similar to that of articular chondrocyte cells, but they are embryologically different and there are distinctions in the extracellular matrix (ECM) produced as well [5,40].

Nutrients, oxygen, and glucose diffuse from the capillary bed through the CEPs, while metabolic wastes diffuse in the reverse direction. Since the NP is avascular, it has the lowest levels of nutrients and pH and the highest concentration of metabolites and osmolarity [5,15].

### 2.2. Annulus Fibrosus

The AF is a fibrocartilaginous tissue comprised of 15–25 layers in which collagen fibers are aligned [11]. This multi-lamellar structure of AF encloses the NP, at the center of IVD, and connects to adjacent vertebrae through the CEPs [15,27]. The AF consists of 65–70% of water, 50–70% of multiple types of collagens and 20% of proteoglycans in dry weight [42,43,44]. These compositions confer IVD with tensile strength supporting compressive and shear stress during complex motion [16,42,43,45,46].

In the hierarchical structure of the AF, the thickness of the layer and the type of collagen vary from the outer AF to the inner AF. The layers become thicker towards the inside, and the anterior and lateral layers are thicker than the posterior layer where lamellae are packed more tightly [11,34,47,48]. Within the AF, the content of collagen type II increases and that of collagen type I decreases when the collagen layer is closer to the NP, whereas the content of collagen type II decreases and that of collagen type I increases when the layer is closer to the outer AF [9,33,49,50,51,52]. Collagen type II, mainly in hyaline cartilage, comprises the NP and the inner AF while contributing to withstand compressive load transferred from the NP and restrain the NP swelling pressure [53,54,55]. Collagen type I, which is highly abundant in fibrocartilage, is stiffer than collagen type II and provides tensile strength [16,42,43,56]. Thus, the tensile modulus of the AF varies by location, and ranges from 59 MPa at inner AF to 136 MPa at outer AF [57]. Collagen fibers are aligned in an inclined structure at each lamella of the AF, with an average angle of 30° varying from 20° to 55° to the transverse plane, and the direction of the oriented collagen fibers is opposite for each alternating layer [11,58,59].

The AF tissue is mostly avascular and aneural and the density of cell is approximately 5–10 × 10^6^ cells/ml [60]. For outer AF, cells are supplied by capillaries and nerve fibers of thin soft tissue, and inner AF cells are supplied through vertebral blood vessels [60,61]. The AF cells have fibroblast-like morphology and the characteristics of both fibroblast and chondrocyte [62].

### 2.3. Cartilage Endplate

The CEP is a thin layer of hyaline cartilage, which binds the NP and AF to the vertebrae [14,15,63]. The thickness of CEP is about 0.5–0.6 mm and decreases with age, especially, the central region that covers the NP [64,65]. The CEP consists of water, collagen type II, and proteoglycan; the collagen content is higher than proteoglycan content [33,64]. The type II collagen fiber network of the CEP and the AF covers the whole NP, supporting the IVD against compressed NP and other complex loads [33,64,66]. Peripheral capillaries penetrate the CEP, causing nutrients to diffuse from vertebral blood vessels into the IVD [15,63,64,67].

### 2.4. Degenerative Disc

Since IVD is a highly hydrated load-bearing tissue, the external pressure or reduction of water content in IVD components cause IVD disorder [9,68]. The condition that the damaged disc results in loss of IVD function is referred to as degenerative disc disease (DDD), and DDD is accompanied with pain from a painless state to an extremely painful state and up-regulation of pro-inflammatory cytokines [69,70,71]. Herniation is a common disc disorder of disc extrusion starting from disc bulging [53,72]. Treatment methods that have been used (e.g., spinal fusion, discectomy, suture) have several limitations such as a poor prognosis, re-herniation, uncomfortable motion, inflammation and persistence of the defective site after treatment [53,73]. Therefore, tissue engineering therapies are required for fundamental treatment and elimination of the risk of recurrence.

## 3. Treatment for Degenerative IVD

In strategies to regenerate degenerative IVD, mimicking both biological and mechanical properties as well as the macro- and micro-structures of the native tissue enhances the biomechanical properties of the materials to facilitate biological and functional regeneration of IVD [74]. Therefore, a number of methods and materials have been studied and used for regeneration of degenerative IVD. Functional polymers used in tissue engineering are appropriate materials to repair and regenerate degenerative IVD because of their great mechanical and biological properties, and capability for proper forms such as hydrogel, particles, and fibers. The properties of polymers and their applications for IVD repair and regeneration are explained in the next section and Table 1. In addition, methods of repairing degenerative IVD include mimicking the macro- and micro-structures enabling immediate treatment for endogenous regeneration. We discuss the promising techniques and investigations in tissue engineering using functional polymers for injured and degenerative IVD.

### 3.1. Functional Polymers for IVD Regeneration

#### 3.1.1. Natural Polymers

• Chitosan

Chitosan is a linear cationic polysaccharide formed from deacetylation of chitin which is a natural component of crustacean and insect exoskeleton [75]. Chitosan which has been approved by Food and Drug Administration (FDA) is a compatible biomaterial because of its non-cytotoxicity, biocompatibility, biodegradability, and antibacterial characteristic [75,76]. Also, chitosan has amino groups and secondary and primary hydroxyl groups for the copolymer synthesis [77]. Chitosan is a temperature and pH responsive hydrogel and is soluble in weak acid and low temperature [77,78,79]. Thus, there are many chitosan-based hydrogels to treat IVD, especially the NP, because of thermoresponsive characteristic of chitosan for less invasive treatment. However, hydrogel consisting solely of chitosan has low mechanical properties compared to the native NP [80]. Thus, other polymers are blended with chitosan to improve biological and mechanical properties of chitosan.

In a study of injectable hydrogel for IVD regeneration, chitosan-based hydrogel was prepared and chondroitin-6-sulfate, collagen type II, gelatin, and fibroin silk were added to facilitate stabilized and biocompatible injectable hydrogel [119]. This hydrogel which targeted the NP simulation started gelation at 37 °C and the gelated hydrogel showed mostly constant storage modulus over the large strain range (0.1–10,000%) in vitro.

For easy inject of chitosan hydrogel, chitosan/poly (γ-glutamic acid) (γ-PGA) nanocomplexes has the advantage in injection process using liquid solutions rather than a highly viscous hydrogel solution [120]. And chitosan/γ-PGA nanocomplexes promoted recovery of IVD native matrix by self-assembly through electrostatic interactions in bovine IVD ex vivo experiment. As a cell culture medium supplement, γ-PGA increases cartilaginous ECM production of mesenchymal stem cells (MSC). Injection of chitosan/diclofenac/γ-PGA nanoparticles reduced pro-inflammatory mediators (IL-6, IL-8, and PGE2) in a degenerated IVD organ model [121].

Thermosensitive hydroxybutyl chitosan (HBC) used as an injectable carrier for encouraging a biologically related reconstruction of the degenerated disc [122], thus, HBC gels can be used as biological agents for enhancing restoration of degenerated disc. N-hexanoyl glycol chitosan (HGC) is an injectable thermosensitive hydrogel, which is synthesized by N-hexanoylation from glycol chitosan and hexanoyl anhydride. HGC hydrogel changes from sol to gel state at 25–56 °C. HGC hydrogel was injected into the NP from a porcine ex vivo model and its gel stability, injectability, and biocompatibility was determined for longer than 28 days [123].

Chitosan with β-glycerolphosphate solution remains in the liquid state with pH level from 6.8 to 7.2, and the chitosan/β-glycerolphosphate gelate at 37 °C [124], because β-glycerophosphate increases pH of solution while inhibiting immediate gelation or precipitation [125]. Bovine NP cell can be cultured in the chitosan/β-glycerophosphate gel, and β-glycerophosphate is used in cell culture medium [126]. 3D chitosan/β-glycerophosphate hydrogel scaffold induced differentiation of human MSC and production of cartilaginous ECM without exogenous molecules [127]. As in the above studies, the distinct phase change feature of chitosan/β-glycerophosphate hydrogel enable the use of injectable scaffold for tissue engineering [128,129].

The major limitations of chitosan hydrogel are relatively low mechanical properties as compared to the native IVD and difficulty of handling. The poor mechanical properties can be overcome by blending or incorporating other polymers to achieve improved mechanical properties. However, problem of handing needs further investigations to make it applicable for practical uses.

• Collagen and Gelatin

Collagen is highly abundant fibrous protein in the human body, and the major structural element of ECM in tissues, especially in the connective tissues [81,82]. Collagen has biodegradability and a better biocompatibility than other natural polymers, but has weak antigenicity [83]. Collagen matrices allow good cell adhesion and proliferation because of an Arg-Gly-Asp (RGD)-like sequence in the protein; collagen is also an excellent surface-active agent that has the ability to penetrate lipid-free interfaces [84]. Gelatin is derived from thermo-treated collagen and is a heterogeneous and water-soluble protein of large molecular weight. Gelatin type A is produced from acid-cured tissues which are pretreated under conditions of pH 8–9 and gelatin type B is produced from lime-cured tissues which are pretreated under conditions of pH 4–5 [74,130,131]. Like collagen, gelatin has good biodegradability and biocompatibility and both collagen and gelatin induce cell adhesion and proliferation and affect collagen type II protein expression [132,133]. Also, both materials have been widely applied in medical uses. Especially, collagen is used for a drug or protein delivery system as various forms of hydrogel, film, sheet, sponge, or nanoparticle, and used for reconstructive surgery as skin, bone or cartilage substitutes [81,134,135,136].

Ovine AF cells seeded on a gel showed better efficacy by adopting high-density collagen gels in in vivo degenerated rat-tail model [137]. To avoid re-herniation and degeneration of AF, high-density collagen gel was crosslinked by riboflavin to tighten the collagen scaffold and improves the viability of fibroblast and chondrocyte [138,139,140]. The high-density collagen gel containing AF cells presented better recovery and NP hydration than the acellular gel at the AF defect site.

Contraction of collagen gel facilitated self-assembly and aligned tissue-engineered AF and IVD [141]. Collagen gel including ovine AF cells surrounded alginate artificial NP. Where cells were dense, collagen fibers were rearranged, creating a circumferential collagen fibril and inducing the formation of a cellular alignment. Unlike the disc structure, the aligned fibrils were well formed in the annular structure and formed better in 1 mg/mL gels than 2.5 mg/mL gels. However, for practical clinical uses, poor mechanical properties and mimicking complex structure of native AF tissue were remained problems.

Gelatin scaffold for MSC transplantation treated on punctured rabbit AF showed effects on the apoptosis and disc height index (DHI) [142]. Transplantation of MSC-pure fibrinous gelatin-transforming growth factor-beta 1 (PFG-TGF-β1) inhibited apoptosis and DHI reduction and increased collagen type II content in defected AF site.

In many cases, gelatin and collagen were used as an additional composite with other materials to improve biomechanical and metabolic properties [85,86,87]. Human AF cells have good adhesion and increased anabolic activity in fibronectin, collagen, and gelatin substrate. The combination solutions of fibronectin (1.7 μg/mL) and collagen (1.3 μg/mL) showed the best cell activity and phenotype [133]. Gelatin and oxidized hyaluronic acid mixture was studied for injectable NP implant after discectomy [143]. In this study, the range of NP motion was restored to normal level, but defective AF remained as a limitation.

• Alginate

Alginate, referred to as alginic acid or algin, is a natural anionic polysaccharide polymer derived from brown algae. It is linear block copolymer comprised of (1,4)-linked β-d-mannuronate (M) and α-l-guluronate (G) [88,89]. Advantages of alginate are biocompatibility, low cytotoxicity, low cost, and easy gelation upon addition of divalent cations (e.g., Ca^2+^, Ba^2+^, Mg^2+^) [89,90]. In order to improve mechanical properties, ionic cross-linking using divalent cations and covalent cross-linking using amine or acryl functionalized crosslinker can be used [91,92,93]. Alginate is a promising biomaterial in wound healing, pharmaceutical applications or drug and protein delivery [144,145,146,147,148]. In addition, alginate hydrogel is known to effective matrix of chondrocyte or NP-like cells [149,150].

In the studies using alginate hydrogel, in situ alginate gelation adopting crosslinkers of carbonate (CaCO_3_) and glucono-δ-lactone were investigated for ex vivo injection into degenerated IVD of bovine caudal spine [151]. Alginate which was tailored for injection to degenerated NP restored disc height and increased the modulus compared to the intact specimen. Optimized hydrogel showed compression modulus of 648.41 kPa and 20.49 kPa on day 1 and 28, respectively. Swelling property of the hydrogel was exhibited by average weight change which is −1.6 ± 6.5% for 24 h. For in vitro and in vivo experiment using alginate-based hydrogel for 3 months, the total tissue-engineered IVD was constructed out of the artificial NP made of alginate-based hydrogel and AF made of electrospun multilamellar matrix polycaprolactone (PCL)/poly (d,l-lactide-co-glycolide) (PLGA)/Collagen type [152]. Alginate hydrogel-based engineered NP were injected to the center of engineered AF. The alginate hydrogel-based NP demonstrated high cell viability of embedded NP cells. The engineered total IVD showed up to 158.71% elongation capability and 1394 MPa of Young’s modulus that supporting shape maintenance and flexibility.

To culture IVD cells especially nucleus pulpous cell in 3D scaffold, alginate hydrogel beads under pressured system were used [153,154]. In the pressure loading system, MSCs were cultured in the 3D alginate hydrogel using poly (ethylene glycol) diacrylate (PEGDA) microcryogels [155]. This system produced similar condition to NP tissue and induced MSCs differentiation to NP-like cells. Moreover, safranin-O staining revealed enhanced glycosaminoglycans (GAGs) production in the pressure group. However, many dead cells were observed because of bad mass transfer between microcryogels and large volume of the matrix.

• Hyaluronic acid

Hyaluronic acid (HA) or hyaluronan is a linear anionic polysaccharide which comprises repeating units of alternated β-1,4-d-glucuronic acid and β-1,3-N-acetyl-d-glucosamine [94]. It is nonsulfated glycosaminoglycan found in ECM throughout the human body, especially in cartilage tissues [95], and can be degraded by hyaluronidases in human [96]. Also, HA can retain water and become viscous formation which is important in homeostasis and biomechanical conditioning of tissues. In the high concentration of HA solution, repulsion of carboxyl groups in HA chains makes the swelling pressure, which leads resilience and malleability [97]. These properties confer HA scaffold stiffness similar to that of native NP [98]. Moreover, HA has biological advantages as a scaffold material. HA binding with the specific cell surface receptors induces transduction of intercellular signals [99]. In addition, interaction between HA and other extracellular molecules helps cell migration and tissue development [97,100,101]. These determine intimate relationship between HA and the cell.

HA hydrogel is formed by addition, condensation reactions or photo cross-linking, and has wide application for drug or cell delivery, and surgical procedures [102,103,104]. Fibrinogen-hyaluronic acid (FBG-HA) hydrogel 3D beads were cultured with bovine NP cells in vitro and in a nucleotomized ex vivo organ model [156]. In vitro NP cell culture in FBG-HA hydrogel showed cell encapsulation efficiency and good stability of gel shape and volume, and a degree of gene expression of NP markers, CA12 and KRT19, remained in the culture. The 2 weeks of ex vivo organ culture using the FBG-HA hydrogel under loads showed 4.2 ± 1.1% of disc height recovery and better integration with the native NP tissue than fibrin gels.

HA has anti-inflammatory properties, analgesic effects [157,158], and its effect on regeneration ability of NP cells were determined [159]. When HA was used for inflammatory cytokine interferon α2β (IFNα2β) inflamed and AF defected bovine IVD, aggrecan and collagen type I synthesis increased and ADAMTS4 which degraded the inflamed matrix was downregulated [160].

#### 3.1.2. Synthetic Polymers

• Polyethylene glycol (PEG)

Polyethylene glycol (PEG) which is referred to as polyethylene oxide (PEO) or polyoxyethylene (POE) is a synthetic polyether. PEG can be synthesized by the reaction of ethylene oxide with water in the presence of a catalyst. Instead of water, ethylene glycol or ethylene glycol oligomers can be a starting material to obtain PEG chain with a narrow distribution of molecular weight [112]. PEG or PEG-based hydrogels have been used clinically for a long time and are commonly used as biomaterials due to their biocompatibility, non-cytotoxicity and easy process of synthesis [161]. PEG-based hydrogels have high hydration and nonionic properties [105]. PEG has no functional groups in the polyether chain except one functional group at the end of the polymer chain. Since PEG has low biorecognition of cells, an additional physical or chemical process is required for enabling cell adhesion and proliferation [106,107].

The multiple PEG-HA hydrogels which were injectable and in situ formable had different effects on morphology and behavior of IVD cells [162]. The PEG-HA hydrogel composed of lower-molecular-weight HA showed higher sulfated GAGs content that was associated with proliferation of IVD cells seeded on the PEG-HA hydrogel.

The hydrogel of photo-crosslinked poly (ethylene glycol)-laminin 111 (PEG-LM111) was developed for regenerating the NP [163]. Laminin induced cell adhesion and biosynthesis by interaction with NP cells. PEG-LM111 hydrogel treated porcine NP cells showed cell clustering, GAGs production, and NP cell metabolism and morphology in both 2D and 3D conditions. For the mechanical properties, torsional shear stiffness of PEG-LM111 were enhanced along with an increase of PEG-diacrylate (PEG-DA) and LM111 concentration. And dynamic shear moduli of PEG-LM111 hydrogel was controllable to that of intact human native NP (7–21 kPa).

Hydrogel made of albumin crosslinked PEG including human IVD cells were experimented in in vitro and in vivo [164]. PEG hydrogel containing IVD cells showed cartilage and IVD specific mRNA, and cell morphology was more stable than monolayer cells. In 2 weeks of in vivo experiments, mouse implanted hydrogel showed more GAGs and collagen than in vitro, and collagen type I and II and aggrecan was expressed in areas where human IVD cells were dense, in addition, ingrowth of connective tissue was observed in the avascular site.

PEG polymers have been studied for cartilage treatment by mimicking cartilage tissue ECM [107,165]. Chondrocytes encapsulated PEG hydrogel containing exogenous HA and DHLSDNYTLDHDRAIH (Link-N) matrix promoted regeneration of cartilage and neo-tissues [165]. Under complex loads like IVD environment Link-N assisted in entrapping HA and GAGs. Link-N is a N-terminal peptide from link protein which is obtained in native IVD and cartilage stabilized aggrecan and HA [166], and HA induced accumulation of aggrecan and collagen type II. This PEG hydrogel incorporating Link-N and HA improved matrix retention, regeneration of cartilage and neo-tissues.

An injectable hydrogel for elastomeric tissue scaffold was prepared by blending linear collagen and PEG [107]. The linear chain of collagen interpenetrated through the networked PEG chain to induce the physical entanglement between the networks. These semi-interpenetrating polymer networks (IPNs) improved viscoelasticity and cell proliferation. At the same PEG concentration (10%), PEG/collagen semi-IPNs and PEG hydrogel showed Young’s modulus of 267 ± 8.8 Pa and 226 ± 12 Pa, respectively.

• Poly-ε-caprolactone (PCL)

Poly-ε-caprolactone (PCL) is a synthetic polyester that is biodegradable and approved by FDA and European Commission (CE) for the use in the human body [12]. PCL is degraded much slower than other materials such as PLGA through hydrolysis, and the decomposition time is controllable by blending with other biodegradable polymers such as poly (vinyl alcohol) (PVA) or poly (glycolic acid) (PGA) to the PCL polymers [108,109]. As a biomaterial, PCL is easy to control mechanical properties and has chemical versatility and high elasticity [110,111]. Although PCL-based scaffolds are usually implanted in a pre-shaped solid form, the scaffolds can be used as hydrogel by combining with other polymers, enabling the scaffolds to function for a longer time [112,167]. PCL can be formed of di- or triblock amphiphilic copolymers by associating with natural or synthetic ones such as PEG, poly (acrylic acid), or poly (N-isopropylacrylamide) (PNIPAAm) [168,169].

Since PCL has been widely used for electrospun nanofibers to mimic aligned fibers of AF tissues, studies on AF treatment are more advanced than NP [12,170].

Alternated angle of fibers in native AF was simulated using multilayered electrospun PCL fibers and the PCL fibrous scaffolds were implanted into rat caudal spine for disc replacement [171]. External fixation enhanced motion stability of this system, and poly (ethylene oxide) layers within the PCL scaffold improved cell infiltration to the acellular scaffold.

Aligned electrospun PCL nanofibrous scaffolds showed different shear modulus depending on fiber angle, the aspect ratio, and presence of cells [172]. The 1:2 aspect ratio had high shear modulus at all angles, especially almost two times higher in 30° condition. MSC seeded scaffolds showed much increased shear modulus compared to acellular scaffolds but determined shear modulus was higher than native AF tissues, requiring methods to control the modulus. Collagen, GAGs and DNA contents didn’t show a difference depending on fiber orientation.

IVD cells and human bone marrow mesenchymal stem cells (hMSCs) were co-cultured in the bi-compartmental hydrogel consisting of electrospun PCL nanofibers and alginate hydrogel [173]. IVD cells were placed in the alginate hydrogel and hMSCs in the bi-compartmental 3D scaffold, and this co-cultured 3D scaffold showed increased production of GAGs, aggrecan, and collagen type II compared to the hMSCs single-culture system.

Besides electrospinning, PCL fibrous scaffolds were fabricated in different processes such as wet-spinning [174]. Thickness of 2 mm circumferential 3D scaffolds, consisting of fibers with 15–27 µm diameter aided rabbit AF cell attachment and proliferation. The AF cells grew along with PCL microfibers, and secreted IVD-related ECM. Mechanical properties were increased with cell culture time. After 21 days of cell culture, compressive modulus improved to 0.26 ± 0.03 MPa which was 0.17 ± 0.02 MPa higher than that of control and tensile modulus improved to 11.23 ± 1.89 MPa which was 10.03 ± 1.37 MPa higher than that of control.

• Polyurethane (PU)

Polyurethane (PU) or polycarbonate urethane is synthetic polymers that consists of urethane groups that have biocompatibility, biodegradability and high physical and mechanical properties such as elasticity, tensile and compressive strength [112,113]. PU is used in many fields, including the medical field, because of its simplicity of manufacturing process and wide range of formation control from brittle to tough and in various forms (e.g., plastic, gel, film, electrospun nanofibers). When PU is decomposed in vivo, water and carbon dioxide come out that are less toxic for the human body.

PU has not yet been studied extensively in IVD treatment, but studies using various forms and mechanical properties of PU are being investigated. A salt leaching/phase inversion method was used to fabricate the PU mass transfer devices. In the degenerated IVD, PU devices which were implanted in the punctured AF site improved ECM production and restoration of degenerated IVD [175]. After 7 days of implanting the PU mass transfer devices in the punctured porcine functional spine, disc height, compressive stiffness, and cell viability demonstrated no significant differences between intact and PU device implanted groups. Furthermore, adenosine-5′-triphosphate (ATP) and proteoglycan contents were higher in the PU device implantation group than in the intact group.

PU with silk fibroin (SF) composite hydrogel was fabricated for NP replacement and investigated in an ex vivo experiment using porcine IVD [176]. The PU/SF scaffold was an injectable hydrogel which was formed by in situ cross-linking. The PU/SF injectable hydrogel perfectly filled the whole degenerated area, thereby improved stability and efficiency of the implant. As a replacement for load bearing tissue, the PU/SF hydrogel showed suitable complex shear modulus (10–21.4 kPa) and compressive modulus (0.2–4 MPa) to implant. Furthermore, increasing cell proliferation and non-cytotoxicity were observed.

The PU scaffold fabricated by electrospun nanofibers showed different properties depending on the arrangement of the nanofibers [177]. The PU scaffold with aligned nanofibers had significantly higher elastic and tensile modulus than the scaffold with randomly arranged fibers. But after wetting process, elastic and tensile modulus of the PU scaffold decreased from 45.6 to 8.9 MPa and from 13.8 to 6.6 MPa, respectively. Although the modulus was decreased upon hydration, it was relatively maintained for 4 weeks after hydration and was comparable to that of native AF tissue. Biodegradability investigated using cholesterol esterase revealed 30% loss in mass within 4 weeks, and no cytotoxicity was found in both degraded and non-degraded segments of the PU nanofiber scaffold.

• Polylactic acid (PLA)/Polyglycolic acid (PGA)

Polylactic acid (PLA) and polyglycolic acid (PGA) are synthetic thermoplastic polymers that are biocompatible, biodegradable, and aliphatic polyester and solid materials [114,115]. The ester group in PLA or PGA can be degraded by hydrolysis. Although pure PGA was limited by hydrolytic instability, PGA-based materials became more suitable as biomaterials through the synthesis of copolymers such as PLGA or poly (glycolide-co-caprolactone) (PGCL) which also have advantageous mechanical properties [116,117]. Especially, PLGA which is a copolymer of PLA and PGA is biodegradable and biocompatible, and has also been approved by the FDA for use in the human body [118]. When PLGA degrades through hydrolysis, lactic and glycolic acids are produced which are less toxic to the human body [178]. There are not many studies on IVD regeneration using PLGA, and the majority of the studies focus on NP restoration.

Scaffolds consisting of PLA-coated PGA and alginate were developed for whole-tissue engineered IVD [179]. For tissue-engineered AF, a PLA/PGA composite was fabricated by coating PLA on the non-woven mesh of PGA and tissue-engineered NP made of mixture of NP cells and alginate was placed within the circumferential structure of tissue-engineered AF. The engineered AF carrying AF cells was implanted within the subcutaneous space of mouse and showed collagen type I and II and increased GAGs content.

To treat defective AF, human AF cells cultured in foams which consisted of poly (D,L-lactide) (PDLLA), a PLA racemic mixture, and Bioglass^®^ particle were fabricated as cell carriers [180]. The expressions of sulfated GAGs and collagen demonstrated non-cytotoxicity and promoting cell adhesion and proliferation characteristic of PDLLA/Bioglass^®^ foams. Furthermore, among the collagen types expressed in the PDLLA/Bioglass^®^ foams, collagen type I was dominant compared to collagen type II.

After the acellular and bioresorbable matrices consisting of non-woven PGA and HA were implanted in a degenerated rabbit disc for 12 months, the PGA/HA matrices implanted group showed 51% increase in MRI signal intensity compared to the control group, and cells migrated into the degenerated site, expressing highly increased levels of collagen type II in the area [181].

PLGA scaffolds having various pore sizes were investigated to analyze NP cell proliferation and mechanical properties of the PLGA scaffold [182]. The PLGA scaffolds on which NP cells were seeded were implanted into the subcutaneous space of mice for 4 and 6 weeks. As the pore size of scaffold increased, cell proliferation, GAGs, and collagen contents increased. The optimum pore size of the PLGA scaffold for carrying NP cells and restoring the NP tissue was 180–250 μm.

As a 3D microstructural scaffold for growth factor or cell delivery to treat the degenerated disc, PLGA microspheres were used [183]. One group of PLGA microspheres loading dexamethasone and transforming growth factor β-3 surrounded by heparin/poly (L-lysine) nanoparticles (PM) was fabricated along with another group of PLGA microspheres loading adipose-derived stem cell (ADSC) (PMA). Both the PLGA microspheres groups were used to treat degenerated rat disc models and showed restoration of disc height and high GAGs content. Especially, collagen type II and aggrecan were expressed at much higher levels in the PMA-treated model compared to that in the normal control and PM-treated models.

### 3.2. Tissue Engineering Methods for IVD Regeneration

• Biomaterial glues for AF repair

Disc herniation occurs when NP comes out through a tear in the AF. The AF tear is a common and inevitable injury after NP discectomy, and the untreated torn AF can lead to additional protrusion of NP [184,185]. Therefore, treating the torn part of the AF is important in the overall IVD treatment after treating NP by filling biomaterials or through discectomy. Although the first attempt to treat defected AF was suture which reduced the re-herniation risk, suturing didn’t restore torn collagen fibers within AF tissues [186,187,188]. For that reason, instead of suturing, biomaterial glues that is the least invasive method have been investigated. The AF closure glue requires biocompatibility, strong adhesion to native AF, mechanical properties comparable to native AF to withstand pressurized NP, and easy delivery and fast setting in surgery [189].

Fibrin glue and isocyanate glues have been studied for torn AF tissue restoration. Fibrin is a biocompatible and porous hydrogel and approved by FDA. It combines with fibrinogen and thrombin when polymerized [190]. However, fibrin lacks biomechanical resistance and stability to be used in load-bearing tissue IVD, especially AF, therefore, fibrin is combined with other polymers (e.g., polyurethane) or crosslinker to enhance mechanical properties [190,191]. Genipin is a plant-derived crosslinker used in food dye and has low cytotoxicity [192]. Genipin crosslinked fibrin hydrogel is compatible with chondrocytes in vitro [193], and have similar biomechanical properties to the AF [186,189,191]. Genipin crosslinked fibrin hydrogel is definitely a potential method to repair small or large AF defects, whereas it needs further long-term in vivo and clinical studies for practical use. Isocyanate-terminated glues have similar elastic modulus to native AF and stronger adhesive properties than fibrin glue [194,195]. However, the isocyanate glues show limitation on full restoration of herniation and toxicity to human AF cells [195].

• Nanofibers

In tissue engineering, many investigations for repairing the AF attempt to mimic structure and mechanical properties of the native AF. Early investigations for tissue-engineered scaffold focused on polymers for supporting cell growth and phenotype. Simulating the biomechanical structure of native tissue is important to achieve a biologically and mechanically proper tissue-engineered AF [196]. Since the AF is comprised of aligned collagen fibers, there are a number of approaches to reproduce tissue-engineered AF using nanofibers or self-assembly fibrils [11]. Electrospinning is a commonly used technique to produce aligned polymeric nanofibers and multiple lamellae to mimic the native AF tissue [12,197,198]. Coaxial electrospinning is a modified method of general electrospinning, producing fibers having a core part and a surrounding shell part. Nanofibers from coaxial electrospinning technique exhibit better mechanical strength and interaction between scaffold and tissue through a strong synthetic polymer core and biocompatible polymer [199]. Other methods to fabricate nanofiber scaffold are using collagen or silk fibers. Silk, which are fibrous proteins, has high mechanical properties, biocompatibility, biodegradability and can be regenerated into various forms, fibers, gel, sponges, films for biomaterials [200]. Lamellar scaffolds are facilitated by wound or knit silk fibers [35,196,201]. In addition to mimicking macro environment of the IVD, studies of mimic microenvironment are performed using silk solution aggregation into nanofibrils within a hydrogel [202]. Collagen found in many human soft tissue and connective tissue is one of the major components of IVD tissues, which contributes to IVD withstanding loads [28,203]. Tissue-engineered total disc replacement with circumferentially aligned collagen fibrils reproduce tissue structure and restore the function [204]. A recent study using collagen fibrils demonstrated that tropocollagen molecules form collagen nanofibrils in hydrogel solution at an appropriate pH level [205]. Hydrogel scaffold including collagen nanofibrils exhibited mechanical stability under complex loads in addition to better cell adhesion and activity than only hydrogel scaffold. Like collagen and silk fibrils in hydrogel scaffolds, nanofiber-based hydrogel scaffolds are investigated to improve the mechanical property and cell adhesion and viability simultaneously [206].

• Cell homing

The IVD is an avascular tissue where nutrients are supplied mostly by diffusion [15], therefore, cell density is inevitably low in the whole of IVD [7]. Low cell metabolic activity and avascular environment limit natural healing and self-regeneration of IVD. Cell homing is one of the less invasive therapeutic method which induces endogenous IVD regeneration. In degenerated human IVD, MSCs migrate from the vertebrae to the NP through AF. The IVD cell migration pattern was investigated by 5-bromo-2-deoxyuridine staining or detecting migration of PKH-labeled MSCs within the IVD [207,208,209]. Chemo-attractive molecules, nutrients, loads, or punctures are factors that increases MSC migration [210]. In chemotaxis of IVD, various chemokines and growth factors induce MSC migration by reacting chemokine receptors on the surfaces of MSC [211]. Herniated IVD tissue attracts cells through chemokines such as CCL5/RANTES and CXCL6, which are secreted from IVD, and allows MSC recruitment in vitro and in organ culture [210,212]. Especially in the NP, expression of CCL2, CCL7 and CXCL8 increased in degenerative IVD tissues [213]. The stromal-derived factor-1 (SDF-1)/CXCL12 in IVD culture improved recruitment of resident MSCs [211], and vascular endothelial growth factor (VEGF) promoted cell growth and migration [214]. For better treatment of herniated IVD and tissue regeneration, polymer-combining strategies are widely studied. A non-woven PGA-HA 3D scaffold showed the potential for promoting human AF cell migration and proliferation [215]. Human serum, platelet-rich plasma, and transforming growth factor β-3 were used to stimulate cell migration, and 3D cultured cells showed an increased gene expression of collagen type I. Freeze-dried acellular resorbable non-woven PGA-hyaluronan implants was used for rabbit disc degeneration model and enhanced the disc water content [181,216].

## 4. Conclusions and Perspectives

Tissue engineering has great benefits as a therapeutic strategy against degenerative IVD, ranging from immediate pain relief to fundamental and long-term endogenous tissue regeneration. Functional polymers are important element in tissue engineering and regenerative strategies for IVD regeneration. We reviewed natural and synthetic polymers which have been generally used as biomaterials and tissue engineering strategies. In the beginning, we describe the macro- and micro-structures, functions, and biological properties of the IVD, which are important for biological and functional regeneration of the tissue.

Substantial studies have focused on developing scaffolds with bio-applicable property like biocompatibility and biodegradability as well as strong compression and tensile strength. Among the suggested polymers in this review, natural polymers showed appropriate biological properties for tissue engineering treatment of IVD. In particular, collagen and HA have advantages in cell proliferation and tissue regeneration of the target tissue in that they are constituents of human cartilage ECM. Moreover, they have a great cell adhesion property. Chitosan and HA have antibacterial and anti-inflammatory characteristics which are advantageous as a scaffold. And chitosan hydrogel promises as a less invasive treatment material since it changes phase according to temperature and pH. However, these natural polymers (chitosan, collagen, alginate, and HA) have low mechanical properties compared to the IVD which has high compression and tensile moduli in MPa units. Scaffolds made of each natural polymers alone are insufficient as a tissue-engineered IVD scaffold. Thus, most studies have fabricated scaffolds by physically or chemically combining a plurality of natural or synthetic polymers. Reviewed synthetic polymers have improved physical and mechanical properties of high stiffness and tensile strength than referred singular natural polymer. Also as biomaterials, PU and PLGA are beneficial in less cytotoxicity because they decompose into water, carbon dioxide, lactic acid, or glycolic acid in the body. But some synthetic polymers (e.g., PEG and PLA) have relatively low cell adhesion property, and thus other materials including collagen are added or copolymers are synthesized. Pre-shaped solid polymers such as PCL, PLA, and PGA are inappropriate for less invasive treatment, but promising approaches are possible with various structures (e.g., particles or hydrogels etc.). Among them, PCL has high elasticity and therefore is suitable for mimicking IVD that requires high Young’s modulus. Many studies using PCL have shown positive results, especially in AF mimicking using electrospinning. On the other hand, PU or PLGA are commonly used in medical treatment, but treatments for IVD are not widely studied. Beside PU and PLGA, clinical test data using functional polymers for regeneration of IVD are still insufficient, especially for CEP. Only a few polymers have been approved by the FDA for use in the human body. To overcome this limitation, investigations at both the laboratory and clinical scale are required.

Nevertheless, the regeneration strategies using functional polymers have been sufficiently considered and were found to offer advantages to degenerative IVD treatment and regeneration. This is because of their synergistic effects via the convergence of various methods and different polymers. Fabrication of copolymers and polymer composites or blending different forms of other polymers together can yield a more suitable mechanical properties and a novel platform scaffold with enhanced biological function for better tissue regeneration. In addition, since the size of vertebrae and IVD and the aspects of individual NP extrusion are different in each patient, the various scaffolds with diverse properties of the polymers can facilitate patient-tailored treatment. The functional polymer scaffolds can be applied in IVD modeling for simulation tests or study of biomechanical functions. Since polymers function as protein or drug carriers, they have great potential in application in further tissue regeneration investigation. Numerous attractive methods such as dendrimer, 4D printing of self-organized protein, or spherical liposomes are not yet in use for IVD treatment, but convergence of the techniques to tissue engineering for IVD regeneration will bring more synergistic effects in future studies. Through the fusion of various technologies and materials, functional polymers have definite potential in endogenous regeneration of the mechanical and biological functions of degenerative IVD.

## Figures and Tables

**Table 1:** Summary and properties of polymers for IVD regeneration.

Polymers		Structure	Source or Monomer	Properties	Ref.
Natural polymers	Chitosan	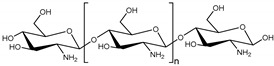	Crustacean and insect exoskeleton	Linear cationic polysaccharide Functional groups: Amino group, hydroxyl group Chitosan and chitosan-based hydrogels are thermoresponsive Hydrogel consisting of only chitosan has low mechanical properties than the native IVD Cell adhesion property is not very good	[75,76,77,78,79,80]
	Collagen and gelatin	Hydroxyproline, proline and glycine are the major constituents of collagen and gelatin.	Animal tissues	Fibrous proteins in the human body, especially in the connective tissues Great biocompatibility than other natural polymers Good Cell adhesion and proliferation of the matrix Used as an additional composite with other materials to improve biomechanical and metabolic properties	[81,82,83,84,85,86,87]
	Alginate	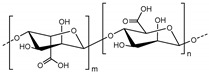	Brown algae	Anionic polysaccharide Functional groups: Carboxyl group, hydroxyl group Easily gelated by divalent cations (e.g., Ca^2+^, Ba^2+^, Mg^2+^) Cross-linking through ionic cross-linking by divalent cations, covalent cross-linking and photo-cross-linking	[88,89,90,91,92,93]
	Hyaluronic acid	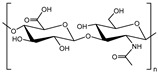 Disaccharides which consist of D-glucuronic acid and N-acetyl-D-glucosamine	Animal tissues, bacteria	Anionic polysaccharide and nonsulfated glycosaminoglycan in ECM of human body Functional group: carboxyl group Biodegraded by hyaluronidases in human body Intimate relationship with cell surface High stiffness than native NP, but low shear force Hydrogels are fabricated by addition or condensation polymerization, or photo cross-linking	[94,95,96,97,98,99,100,101,102,103,104]
				Carbohydrate has aldehyde group generally at the polymer chain end	
Synthetic polymers	Polyethylene glycol (PEG)	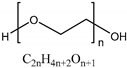	Ethylene glycolEthylene oxide	Nonionic and highly hydrated polymers No functional groups in polyether chain Low biorecognition of cells Used as copolymer or composites to improve its mechanical properties	[105,106,107]
	Poly-ε-caprolactone (PCL)	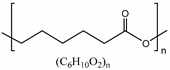	ε-caprolactone	Functional group: Carbonyl group Degradation time is much longer than other materials and controllable Easy process and simplicity of control mechanical properties Ultraviolet/ozone treatment increases PCL surface charge to introducing oxygen functional group Approved by FDA and CE for the use in human body	[12,108,109,110,111]
	Polyurethane (PU)	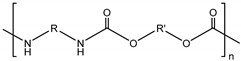	Synthesis of diol and diisocyanate	High physical and mechanical properties Easy to control strength level and formation PU is decomposed to water and carbon dioxide	[112,113]
	Polylactic acid (PLA) and polyglycolic acid (PGA)	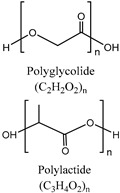	Lactide/Glycolide	Aliphatic polyester Functional group: Ester group, hydroxyl group, carboxyl group Solid materials which are thermoplastic polymers PGA copolymers have advantages in mechanical properties PLGA, a copolymer of PLA and PGA, has FDA approval for the use in human body	[114,115,116,117,118]
